# Editorial: Towards 2030: Sustainable Development Goal 16: peace, justice and strong institutions. A sociological perspective

**DOI:** 10.3389/fsoc.2025.1563951

**Published:** 2025-02-18

**Authors:** Benjamin W. Kelly, Todd O. Smith

**Affiliations:** ^1^Department of Psychology, Sociology, and Childhood Family Studies, Nipissing University, North Bay, ON, Canada; ^2^Association for Baha'i Studies, Ottawa, ON, Canada

**Keywords:** UN Sustainable Development Goal 16, resource management, public sphere, social solidarity, prosocial behavior, equitable institutions

This Research Topic focuses on the United Nations Sustainable Development Goal 16 (UNSDG 16), which aims to “build effective, accountable and inclusive institutions at all levels” (United Nations, n.d.). The four articles in this Research Topic present global contributions from various disciplines and insights from the social sciences. The editorial highlights the interconnected challenges related to achieving UNSDG 16, emphasizing the need to develop more accountable institutions that ensure equitable access to justice for all citizens.

Using the Mackenzie Gas Project as a case study, Dokis examines the challenges of achieving UN Sustainable Development Goal 16 in the context of emerging co-management systems between Indigenous communities and corporate-state actors. The author illustrates how exploitive social structures rely on an extractivist resource management logic that often prioritizes measurable economic gains while ignoring latent relational harms faced by Indigenous communities. Although technological remediation and impact benefit agreement initiatives are essential, Dokis is demonstrates that these refinements cannot adequately address the subtle and intangible challenges faced by these communities. To truly foster inclusivity, Dokis's ethnographic research suggests the need for an all-inclusive transformation that dismantles the deep-rooted systems of colonialism, power dynamics, and social inequality mirrored in these institutions and their problematic environmental assessments. This study reminds us that progress toward inclusivity can only be achieved through a collaborative and thoughtful re-examination of authority.

Utilizing the Habermasian social theory of the public sphere and drawing on a Bahá'í-inspired model of democratic governance, Sabet explores the possibility of an alternative prefigurative politics that promotes and sustains a sense of purposefulness tethered to a unified humanity and an overarching narrative of human history. Given the many challenges of individualistic governance, he asks how “—given the dynamics of lifeworld and system…can communicative action be reliably and sustainably fostered within the liberal democratic state” (p. 3). Sabet turns to the Bahá'í community, arguing that it exemplifies the creation of effective, accountable, and inclusive institutions. This approach is not characterised by a naïve optimism but rather by a practical, empirically based framework grounded in consultation and collective decision-making. It aims to move away from narratives centred on self-interest and instead encourages, promotes, and celebrates the nobility of humanity and our shared purpose.

Hedley's “prosocial protagonists” framework emphasizes the role of collaborative change agents who work alongside diverse stakeholders to advance shared human interests. These actors include individuals, communities, and institutions. Discerning these relationships of mutual influence is crucial in identifying effective collaborative strategies. Hedley's perspective recognizes the complexities of human motivation and stresses the essential role of social impact, leadership, and teamwork in fostering prosocial behaviours. Additionally, it aims to move beyond simplistic interpretations of human nature and promote a more sophisticated understanding of motivation and collaboration that is conducive to achieving the UN development goals.

Sayuti et al. provide compelling empirical evidence on the variable effects of prosocial behaviour and social solidarity during the COVID-19 pandemic in Indonesia. Notably, the research reveals that rural areas participated in significantly higher levels of “mutually beneficial cooperation” during the pandemic than urban areas. Rural communities benefited from well-connected social networks and longstanding traditional practices already established before the pandemic, unlike urban localities, which were not as well buffered because these forms of social solidarity did not exhibit similarly robust forms of information-sharing and resource-sharing activities during COVID-19. These findings speak to the tensions and dilemmas governments encounter when trying to balance health protocols, such as social distancing, with the negative impact that enforcing these rules can have on social solidarity. The results emphasize the importance of clarifying contextual factors in expressing diverse forms of social solidarity and planning regionally appropriate health policies. A nuanced comprehension of these dynamics reveals the variation in exposure to COVID-19 and the factors contributing to mortality. Furthermore, this research identifies diverse socio-cultural factors in implementing preventive measures and coordinating collective responses to challenges and crises while simultaneously paying close attention to the location of residents and designing safeguards so that these communities do not experience an erosion of their social solidarity.

Achieving Sustainable Development Goal 16 requires a universal multi-dimensional approach. The four articles constituting this Research Topic, are authored by dedicated applied researchers and active practitioners who are committed to developing solutions that establish fair public processes and promote transparent social frameworks. These efforts aim to enhance the capabilities of individuals, communities, and international stakeholders by unleashing their creative potential and working to promote, foster, and co-create effective and inclusive participatory decision-making at all levels. This interdisciplinary approach to attaining UNSDG 16 considers the cultural, social, political, institutional structures and economic factors that influence human behaviour and societal responses to crises. To move away from individualistic and exploitative models, it embraces prosocial values, builds authentic partnerships, encourages inclusive engagement, and recognizes the importance of context-specific methods. The conceptual frameworks and empirical findings presented in these editorial contributions offer valuable insights for implementing transformative change and ultimately contribute to a deeper appreciation for cultivating more cooperative, inclusive, equitable and flexible institutions.
